# Testing a Smartphone-Based Intervention Targeting Anxiety Sensitivity Among Women Presenting for Emergency Care After Sexual Assault: Pilot Randomized Controlled Trial

**DOI:** 10.2196/86612

**Published:** 2026-05-25

**Authors:** Nicole A Short, Regine Deguzman-Lucero, Rachel Weese, Natalie Chisam, Jenny Black, Karen Serrano, Samuel McLean

**Affiliations:** 1 Department of Psychology, University of Nevada, Las Vegas, Mail Stop: 5030 4505 S. Maryland Pkwy, Las Vegas, NV, 89154, United States, 1 7028950606; 2 SAFE Austin, Austin, TX, United States; 3 Department of Emergency Medicine, University of North Carolina at Chapel Hill, Chapel Hill, NC, United States; 4 Department of Psychiatry, University of North Carolina at Chapel Hill, Chapel Hill, NC, United States

**Keywords:** anxiety sensitivity, feasibility, randomized controlled trial, sexual assault, posttraumatic stress

## Abstract

**Background:**

Anxiety sensitivity (AS), defined as the fear of anxious arousal, is a promising therapeutic target for reducing posttraumatic stress disorder (PTSD) symptom development after trauma exposure. Initial research suggests that smartphone-based AS interventions may be acceptable to sexual assault survivors at risk for PTSD symptoms and effective for symptom reduction, but only small one-arm proof-of-concept studies have been conducted.

**Objective:**

The goal of this study was to extend prior proof-of-concept work by conducting a pilot randomized controlled trial. The aims were to evaluate intervention efficacy, AS and PTSD symptom change, the acceptability and credibility of a control intervention, and the feasibility of a larger randomized controlled trial.

**Methods:**

A total of 60 women with high AS presenting for emergency care after sexual assault were recruited and randomized to either the AS intervention or a control condition, and they were followed up with for over 6 months via remote self-report questionnaires.

**Results:**

The findings indicated that the study population is at risk and in need of intervention: 88.8% (40/45) and 80.6% (29/36) of women sexual assault survivors in the sample met the criteria for probable PTSD at 7 weeks and 6 months post assault, respectively. Most (16/27, 59.3%) individuals receiving the AS intervention who completed it rated it as acceptable (eg, 18/21, 85.7% reported that the treatment was helpful). Early within-group reductions were not statistically significant, but by month 6, statistically significant reductions in AS and PTSD symptoms were observed in both conditions. Recruitment and retention data supported the feasibility of the study design, although some suggestions were noted for future research (eg, improving intervention and ecological momentary assessment compliance).

**Conclusions:**

This pilot study replicated the proof of concept and acceptability of a novel smartphone-based intervention targeting AS delivered to women sexual assault survivors presenting to emergency care. Intervention completion was in line with or better than traditional therapy but remained a challenge. Our selected control condition demonstrated a larger effect than expected, and we were unable to track its initiation or completion, causing difficulty in drawing conclusions. Overall, the results highlight the need for additional research.

## Introduction

Approximately 100,000 women annually seek emergency care after sexual assault in the United States alone [1]. More than half of these women develop persistent posttraumatic stress disorder (PTSD) symptoms; however, the vast majority do not receive any additional health care services outside of the emergency care visit [2]. PTSD and commonly comorbid symptoms, including anxiety, depression, pain, and substance use, impose a large burden of suffering on sexual assault survivors [3]. In light of both the personal suffering experienced by sexual assault survivors and the substantial societal costs (estimated at US $2.8 trillion over the lifetimes of women survivors [4]), the development of scalable secondary preventive interventions to reduce PTSD development after sexual assault is a public health priority.

One important cognitive process implicated in PTSD development after sexual assault is anxiety sensitivity (AS). Individuals with elevated AS, or fear of anxious arousal [5], may interpret normative posttraumatic reactions, such as intrusive memories, physiological reactions to trauma reminders, and hypervigilance, as threatening and something to be avoided [6,7]. Consistent with this theoretical notion, AS has been empirically demonstrated to be elevated among those with PTSD and to prospectively predict PTSD onset [8,9], course [10], and severity [11], including among sexual assault survivors presenting for emergency care [12].

Importantly for preventative intervention development, AS can also be modified by intervention. Brief computerized cognitive behavioral interventions have been shown to reduce AS [13,14], and, in turn, PTSD [15-17] and related symptoms such as anxiety and depression [13,14]. These interventions provide psychoeducation, including the adaptive nature of anxiety, and cognitive behavioral strategies, such as cognitive restructuring and interoceptive exposure. Such interventions have been demonstrated to be acceptable to clinical samples of community individuals with elevated AS [18].

Recently, we developed and conducted initial acceptability and feasibility testing of an AS intervention (RISE [RCT for Innovating Stress-Related E-Health] Guide) to reduce the development of PTSD in adult sexual assault survivors [19]. The RISE Guide uses methods with demonstrated use in previous AS interventions, including psychoeducation, cognitive restructuring, and interoceptive exposure. However, the content has been modified to be smartphone-based and specifically tailored to target PTSD symptoms (eg, psychoeducation is focused on the stress reaction after trauma and its functional use). Furthermore, the audio-visual and interactive content of the RISE Guide includes an ecological momentary assessment or intervention [20] component, which provides as-needed reminders of intervention concepts. This novel AS intervention was tested for proof of concept and acceptability among 12 women seeking emergency care after sexual assault [19]. The results indicated that 75% of participants initiated the intervention, with the majority (66.6%) completing most of it [19]. Participants rated the RISE Guide as logical and useful, perceiving it to be potentially helpful in reducing their symptoms [19]. In addition, the results showed moderate-to-large reductions in both AS and PTSD symptoms over 6 weeks post assault [19]. Therefore, AS interventions in general, and this specific, newly developed intervention, show promise as potential scalable interventions to be provided to women seeking emergency care after sexual assault.

The ORBIT model of behavioral intervention development offers a systematic framework to guide the development and testing of promising behavioral interventions to prevent chronic diseases [21]. Specifically, following the acceptability and proof-of-concept evaluations (phases 1 and 2a) [21], such as those described above [19], the next step in assessing promising interventions, such as the RISE Guide, is conducting a pilot study (phase 2b). A pilot study is designed to replicate the findings of clinically significant benefits from the active intervention and to assess the adequacy of the chosen control group prior to moving on to a pilot feasibility trial (phase 3). Of note, this study was initially designed as a “preliminary efficacy trial,” but given increasing recognition of the problems with these types of designs [22-25], it was shifted to a phase 2b pilot study. Therefore, this study was designed to address these next steps in intervention development by replicating proof-of-concept testing of the novel AS intervention (“RISE Guide”) and evaluating (1) intervention acceptability, (2) behavioral risk factor (AS) and symptom (PTSD) change, (3) acceptability or credibility of the chosen control intervention (“Breathe2Relax” [B2R], a digital app teaching relaxation techniques such as diaphragmatic breathing) and its impact on AS [26], and (4) the feasibility of a larger randomized controlled trial (RCT) testing the novel AS intervention among women presenting for emergency care after sexual assault (ie, whether 3‐4 participants per month could be recruited, >70% of the sample could be retained, and >70% would complete the RISE Guide).

## Methods

### Recruitment

Participants (N=60) were recruited from emergency care sites within the Better Tomorrow Network from July 2021 to May 2022 (NCT05305235). Patients were eligible for the study if they met the following inclusion criteria: (1) identified as a woman (both natal and self-identifying); (2) were 18 years or older at the time of emergency care; (3) presented within 72 hours of the sexual assault; (4) self-identified as being able to speak, read, and write English; (5) owned a smartphone with continuous service for over a year; and (6) reported elevated AS (a score of over 17 on the Anxiety Sensitivity Index-3 [ASI-3] [27]). Potential participants were excluded from the study if they (1) did not meet the criteria for the study, (2) were unable to provide informed consent, (3) were prisoners or incarcerated, (4) were currently pregnant, (5) reported currently living with their assailants with plans to continue doing so (as repeated traumatization would impact recovery), (6) lacked a mailing address, (7) reported that they were currently receiving inpatient care, (8) had previously enrolled in the study, or (9) did not have a sexual assault nurse examiner (SANE) examination. Participants were not excluded if they accessed other mental health care services and were able to do so throughout the trial if desired. All participants gave informed consent at the time of emergency care after receiving a full explanation of the study.

### Ethical Considerations

All study procedures were approved by the University of North Carolina (UNC) Institutional Review Board (20‐3494) and adhered to ethical standards. Each study site relied on UNC’s institutional review board. Prior to study participation, all study participants completed a virtual consent process via Zoom (“teleconsent”) during which they were provided with the documentation of electronic informed consent via DocuSign while on Zoom with a study research assistant. Participants were informed of their study rights, including the right to withdraw from the study, and were provided with an ID number. The data were hosted on the electronic data capture platform, REDCap (Research Electronic Data Capture [28,29]), and all data were deidentified prior to analysis. The data collected for this study were accessible only to trained study staff and stored securely on electronic servers. In exchange for study participation, participants were compensated upon the completion of study activities (eligibility screening [US $40], week 1 survey [US $50], on-time intervention completion [US $50], ecological momentary assessments [EMAs; US $1/EMA], week 7 survey [US $50], contact check-in [US $10], and month-6 survey [US $50]). The study team monitored for adverse events, but none were noted in either condition. Participants’ privacy and confidentiality were protected, and participants are not identifiable in any way in this study.

### Study Design

This is an analysis of a 2-arm, double-blind, pilot RCT evaluating the feasibility of a digital health intervention aimed at reducing AS following sexual assault. Research coordinators were aware of condition assignments; however, the study’s primary investigators were not. Potential participants for this study were referred by SANEs from 2 emergency care sites in the United States, Stop Abuse For Everyone Austin (a community-based human services agency serving survivors of sexual assault, including by providing SANE examinations) and the UNC, Chapel Hill Emergency Department (a SANE program embedded in the Emergency Department at the UNC Hospital System). SANEs at these sites screened all patients presenting for sexual assault for the study using a standardized eligibility form and recorded data regarding eligibility, which was provided to the study team. Of note, participants could meet criteria for multiple exclusion criteria; thus, percentages add up to >100%. SANEs conducted initial eligibility screenings with patients to determine their initial eligibility (ie, adult women with access to a smartphone presenting within 72 hours of sexual assault). SANEs also asked such patients whether they were interested in participating in a research study to determine whether a “new online treatment program” might be effective in improving recovery after sexual assault. If potentially eligible and interested, SANEs called the on-call study research assistant (RA), who initiated a video call with the participant during emergency care to provide information about the study. If it was not possible to conduct this during emergency care, the video call would be scheduled for the next day. During this videoconference, the RA would then provide study information and gauge interest. Those interested in participating were screened for the remaining eligibility criteria (eg, elevated AS) and, if eligible, completed the teleconsent process. If at any point during this process, the participant was deemed to be ineligible or no longer interested, these data were recorded. Following teleconsent, participants completed a survey hosted on REDCap, in which sociodemographic information and baseline self-report measures of AS, PTSD, and substance use were collected.

The day after enrollment through 7 weeks post assault, EMAs and interventions were sent via SMS 4 times a day for 7 weeks. Participants were asked to complete 3 out of 4 surveys per day, with 1 additional survey sent to compensate for any missed surveys. One week postassault, participants received an invitation to complete a survey hosted on REDCap to provide additional data that included measures of AS and PTSD. Those who completed the week 1 survey were randomized by a research coordinator using randomization tables (generated by staff statistician) in REDCap stratified by study site, into 1 of 2 study conditions, B2R, or RCT for Innovating Stress-Related E-Health (RISE) Guide. Those who did not complete the week 1 survey were thanked for their interest, and their study participation was discontinued. Follow-up surveys were also completed remotely through REDCap at week 7 and month 6 post sexual assault. Participants were compensated for all assessments.

### Interventions

#### Overview

In this study, participants were randomized to 1 of 2 study arms, the RISE Guide or B2R intervention. Both study conditions were similarly designed to be accessed via a mobile app and reduce symptoms post sexual assault.

#### RCT for Innovating Stress-Related E-Health

The RISE Guide is a web-based app targeting AS following a recent sexual assault. The RISE Guide, which is hosted on the Qualtrics platform, was adapted from a previously computerized, validated intervention for AS (cognitive anxiety sensitivity treatment) [14]. The RISE Guide was modified to be suitable for smartphones and focused on symptoms of anxious arousal after a sexual assault. The intervention delivers over 40 minutes of psychoeducation and cognitive-behavioral principles in an interactive, audio-visual format. The RISE Guide consists of 3 modules: stress response and common reactions after trauma, myths related to stress, and exposure. Module 1 focuses on the stress response and common reactions after trauma, module 2 discusses myths related to stress, and module 3 consists of a guided interoceptive exposure exercise. Module 3 also encourages participants to approach and engage, rather than avoid safe situations that induce postassault-related fear. To support understanding and maintain attention, users were also quizzed on the information from the modules and received feedback based on responses. Participants were randomized after the completion of the week 1 time point, at which point they could begin accessing the RISE Guide and were asked to complete it within 2 weeks. Also after the week 1 timepoint and randomization, ecological momentary intervention (EMI) support was provided after the completion of week 1 for 7 weeks after the assault as an adjunct to the RISE Guide to support intervention principles. Participants did not necessarily need to finish the RISE Guide before receiving these EMIs; they began after randomization regardless of intervention completion. During these 6 weeks, participants received 3 EMIs per day. EMIs were personalized to provide psychoeducation and/or coping tools based on EMA reports of individual PTSD symptom levels, substance use, or arousal and reactivity symptoms. For example, if a participant reported high re-experiencing symptoms, they would receive a message normalizing their symptoms, and then be linked to review relevant content pertaining to those symptoms. Similarly, if a participant reported high levels of craving, they would receive a message about coping tools. Each EMI took about 2.5 minutes to complete. In total, participants completed up to 105 minutes of study activities. Of note, intervention initiation, progress, and completion can be independently verified via Qualtrics.

#### Breathe2Relax

B2R is a mobile app designed to teach users diaphragmatic breathing to reduce anxiety [26]. Participants watched a demonstration of correct diaphragmatic breathing and learned about stress and its effects through reading or watching videos embedded in the app. Participants then practiced breathing through guided interactive videos. Participants assigned to this condition downloaded the B2R app onto their smartphones and received SMS reminders to use it.

### Measures

#### Sexual Assault Characteristics

Characteristics of the assault were collected from data obtained from the SANE medical or forensic records. All data collected from these records were obtained with participant consent. RAs and a research coordinator were trained to review medical records based on a coding protocol developed by the first author. Each medical record was coded twice by 2 RAs. Discrepancies in coding were reviewed by the first author.

#### PTSD Checklist-5

The PTSD Checklist-5 (PCL-5) is a 20-item self-report measure of *Diagnostic and Statistical Manual of Mental Disorders, Fifth Edition* (*DSM-5*) PTSD symptoms within the past month [30]. For this study, participants rated current PTSD symptoms on a 5-point Likert scale, with instructions modified to focus on symptoms related to their recent sexual assault. The 5-point Likert-type scale response options range from 0 (not at all) to 4 (extremely). Items were summed to create a total symptom severity score ranging from 0 to 80, with higher scores indicating greater symptom severity. Participants were administered the PCL-5 upon receiving emergency care and subsequently at week 1, week 7, and month 6 post assault. The PCL-5 demonstrates strong psychometric properties [31] and, in this study, it demonstrated good-to-excellent internal consistency (*ɑ*=.80‐.95). For this study, given the high PCL-5 scores common after a sexual assault, we selected a more stringent PCL-5 score cutoff criterion of greater than or equal to 45 to indicate a probable PTSD diagnosis [32,33].

#### Anxiety Sensitivity Index-3

The ASI-3 is an 18-item self-report measure of AS or concerns regarding anxiety [34]. Participants responded to items using a 5-point Likert scale ranging from 0 (very little) to 4 (very much). Items were summed to create a total score ranging from 0 to 72. The participants were administered the ASI-3 at emergency care, 1 week (week 1), and 6 weeks (week 6) post sexual assault. An ASI-3 score greater than or equal to 17 indicates moderate anxiety sensitivity [27]. The ASI-3 demonstrates good reliability and internal consistency [34] and, in this study, it demonstrated good-to-excellent internal consistency (ɑ=.88‐.94).

#### Credibility/Expectancy Questionnaire

The Credibility/Expectancy Questionnaire (CEQ) is a 6-item measure of treatment expectancy and rationale credibility [35]. This measure uses 2 different response scales, one 9-point Likert scale ranging from 1 (not at all) to 9 (very logical) and another with response options ranging from 0% to 100%. Item responses were standardized. A composite score for each of the factors (credibility and expectancy) was calculated by summing the items. The CEQ was administered at week 1 post sexual assault. The CEQ demonstrates good reliability and internal consistency [35]. In this study, it demonstrated excellent standardized internal consistency (*ɑ*=.92).

#### Week 7 Study-Specific Acceptability and Use Measure

Seven weeks into the intervention, participants responded to a 10-item questionnaire assessing the RISE Guide intervention website or the B2R app. Six items assessed the frequency of use, ease of understanding, helpfulness, frequency of using information or techniques from the intervention, and whether they would recommend it to others, with a 5-point Likert response scale (eg, 0=completely to 5=not at all; 0=very often to 5=not at all). Four open-ended items assessed app perception (eg, likes, dislikes, what was helpful) and future suggestions for the RISE Guide intervention.

## Results

### Descriptive Statistics

**Figure 1.**
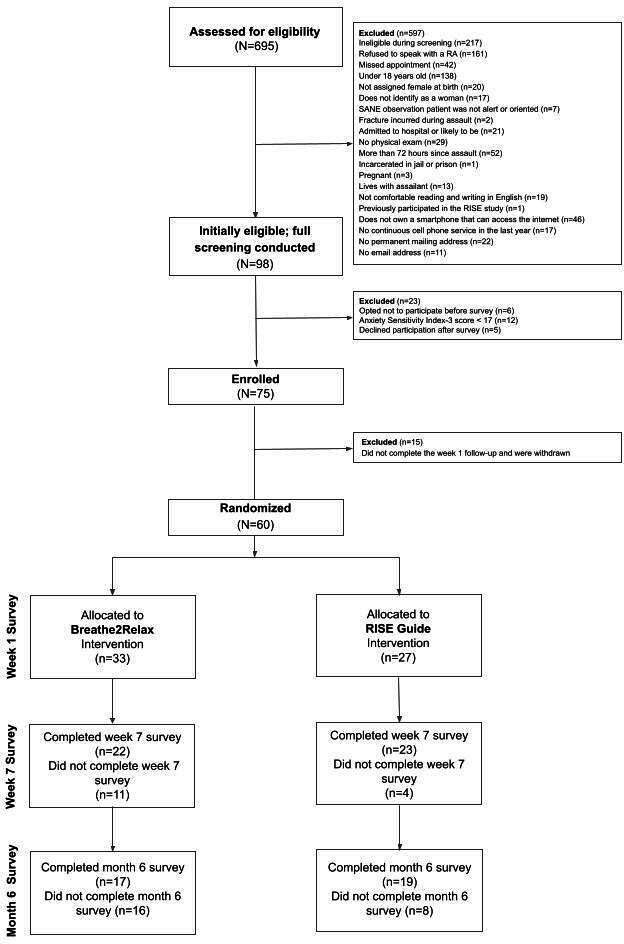
Flowchart of women sexual assault survivors presenting for emergency care and participating in the randomized controlled trial (RCT) for posttraumatic stress disorder prevention. The number of reasons for exclusion exceeds the number assessed for eligibility due to the ability to choose multiple criteria for exclusion. RA: research assistant; RISE: RCT for Innovating Stress-Related E-Health; SANE: sexual assault nurse examiner*.*

Ninety-eight of the 695 sexual assault survivors screened for eligibility at emergency care were determined to be eligible for the study (Figure 1). Of those, 23 declined participation and 75 enrolled in the study. Fifteen participants did not complete the week 1 follow-up survey, were not randomized, and were withdrawn from study participation. The final sample consisted of 60 participants: 33 (55%) participants were randomized to B2R, and 27 (45%) participants were randomized to the RISE Guide intervention.

The demographic characteristics of the sample are included in Table 1. On average, participants were 26.19 (SD 6.72) years old. Participants identified as majority White (37/60, 61.6%). Of the sample, 31.6% (19/60) identified as Latina or Hispanic. A majority of the sample reported having a college degree, an annual income of less than US $60,000, and full-time employment. There were no significant differences in education (*P*=.79), household income (*P*=.07), employment (*P*=.17), relationship status (*P*=.61), race (*P*=.19), or age (*P*=.90) between recruitment sites. However, there was a significant difference in ethnicity between sites (*P*=.04), such that 32.1% (17/53) of Stop Abuse For Everyone Austin participants identified as Latina or Hispanic, while none from UNC did. Regarding clinical characteristics at week 7, 88.8% (40/45) of the sample met criteria for probable PTSD based on a PCL-5 cutoff of 33, and 62.2% (28/45) met criteria using a cutoff of 45. At month 6, this decreased slightly to 80.6% (29/36) reporting a PCL-5 score of 33 and above and 52.7% (19/36) people reporting a score of 45 and above. Zero-order correlations revealed significant concurrent associations between AS and PTSD symptoms, and between reductions in AS and PTSD symptoms from week 1 to week 7, and week 7 to month 6 (Table S1 in Multimedia Appendix 1).

**Table 1.** Demographic characteristics of women sexual assault survivors presenting for emergency care and participating in this randomized controlled trial for posttraumatic stress disorder prevention^a^.

Demographic characteristics	Breathe2Relax (n=33), n (%)	RISE^b^ Guide (n=27), n (%)	*χ*² (*df*)
Race			4.33 (6)
Asian	1 (3)	0 (0)	
Black or African American	3 (9.1)	5 (18.5)	
Native Hawaiian or Pacific Islander	1 (3)	0 (0)	
White	20 (60.6)	17 (63)	
Multiracial	2 (6.1)	2 (7.4)	
Not listed	2 (6.1)	0 (0)	
Preferred not to answer or missing	4 (12.1)	3 (11.1)	
Ethnicity			0.01 (1)
Latina	11 (33.3)	8 (29.6)	
Not Hispanic or Latina	22 (66.7)	17 (63)	
Education (highest level completed)			1.23 (5)
Less than high school education	1 (3.1)	0 (0)	
High school education	4 (12.5)	3 (11.1)	
Some college or post high school training	12 (37.5)	12 (44.4)	
College degree or more	15 (46.2)	12 (44.4)	
Annual income (US $)			8.26 (5)
0-19,999	8 (25.8)	4 (19)	
20,000-39,999	7 (22.6)	9 (42.9)	
40,000-59,999	5 (16.1)	6(28.6)	
60,000-79,999	4 (12.9)	0 (0)	
80,000-99,999	4 (12.9)	0 (0)	
100,000-149,999	3 (9.7)	2 (9.5)	
Work status			2.43 (4)
Unemployed or homemaker or disabled or prefer not to answer	7 (21.8)	8 (30.7)	
Part-time employee	7 (21.9)	7 (26.9)	
Full-time employee	18 (56.3)	11 (42.3)	

a There were no statistically significant differences between Breathe2Relax and RISE Guide participants. Participants who selected “prefer not to answer” were excluded from the demographic characteristics table in the study.

b RISE: Randomized Controlled Trial for Innovating Stress-Related E-Health.

### Assault Characteristics

Characteristics of the sexual assault were collected upon presentation to emergency care. A majority of the participants reported knowing their assailant. Participants identified their assailant as a friend or acquaintance (12/42, 28.6%), someone with whom they had a planned first encounter (eg, first date; 10/42, 23.8%), former romantic partner (1/42, 2.4%), or talking in an undefined relationship (1/42, 2.4%). A smaller portion of participants reported their assailant as a stranger (11/42, 26.2%). Participants reported loss of consciousness during the assault (23/32, 71.9%) and strangulation (14/60, 23.3%). A majority (35/44, 79.5%) of the participants reported penile-vaginal penetration.

### Proof of Concept of the RISE Guide

#### Acceptability

Treatment acceptability was assessed using the CEQ and a measure designed for this study (Table 2). The results of the CEQ indicated that the RISE Guide participants generally had a positive impression of the intervention (mean 33.1, SD 13.2). With respect to the study-specific acceptability measure, the majority (18/21, 85.7%) of the participants found the RISE Guide to be at least somewhat helpful, 95.2% (20/21) reported that it was at least somewhat interesting, 90% (18/20) would at least sometimes recommend it to others in a similar situation, and 95.1% (20/21) indicated that it was at least somewhat easy to understand.

**Table 2.** Study-specific measures of treatment use and acceptability for the Randomized Controlled Trial for Innovating Stress-Related E-Health (RISE) Guide among women sexual assault survivors presenting for emergency care and participating in this randomized controlled trial for posttraumatic stress disorder prevention.

Items and responses	Participants, n (%)
Frequency of using information in treatment (n=18)	
Very often	4 (22.2)
Often	5 (27.8)
Sometimes	4 (22.2)
Occasionally	5 (27.8)
Not at all	0 (0)
Was the information in your treatment helpful? (n=21)	
Completely	7 (33.3)
Mostly	8 (38.1)
Somewhat	3 (14.3)
Slightly	2 (9.5)
Not at all	1 (4.8)
How interested were you in your treatment? (n=21)	
Completely	6 (28.6)
Mostly	7 (33.3)
Somewhat	7 (33.3)
Slightly	0 (0)
Not at all	1 (4.8)
Would you recommend your treatment to other women who recently experienced assault? (n=20)	
Completely	10 (50)
Mostly	6 (30)
Somewhat	2 (10)
Slightly	2 (10)
Not at all	0 (0)
Was your treatment easy to understand? (n=21)	
Completely	12 (57.1)
Mostly	4 (19)
Somewhat	4 (19)
Slightly	1 (4.8)
Not at all	0 (0)

### Proof of Concept

#### AS Outcomes

Multiple imputation was used to account for missing data at later time points. At week 1 post sexual assault prior to randomization, individuals reported elevated AS scores, which were also slightly higher than those in the control condition (mean 44.88, SD 15.88; Table 3), which decreased at week 7 (mean 40.54, SD 16.12). From week 7 to month 6, there was an additional decrease in AS scores (mean 33.57, SD 16.75). By month 6, 2-tailed paired *t* tests with week 1 scores indicated that AS scores had decreased by 11 points (Hedge *g*=0.76). However, a between-groups linear regression analysis indicated that the RISE Guide condition was not a significant predictor of reductions in AS (*P*=.77).

**Table 3.** Comparison of outcome scores across conditions among women sexual assault survivors presenting for emergency care and participating in this randomized controlled trial for posttraumatic stress disorder prevention^a^.

Condition	AS^b^ scores, mean (SD)			PTSD^c^ scores, mean (SD)		
	Week 1	Week 7	Month 6	Week 1	Week 7	Month 6
RISE Guide^d^	44.88 (14.88)	40.54 (16.12)	33.44(16.77)	57.56 (11.96)	52.94 (15.76)	44.11 (17.09)
B2R^e^	40.91 (17.91)	35.67 (16.98)	29.90 (17.92)	57.74 (11.90)	49.04 (17.43)	44.94 (14.87)

a Multiple imputation was used to account for missing data; therefore, all participants were included in analyses.

b AS: anxiety sensitivity.

c PTSD: posttraumatic stress disorder.

d RISE Guide: Randomized Controlled Trial for Innovating Stress-Related E-Health (active AS intervention).

e B2R: Breathe2Relax (control condition).

#### PTSD Outcomes

**Figure 2.**
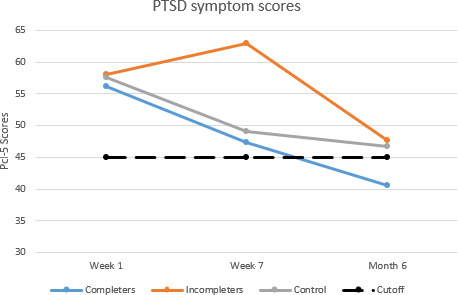
Posttraumatic stress disorder (PTSD) symptom score changes among women sexual assault survivors presenting for emergency care and participating in this randomized controlled trial for PTSD prevention*.* PTSD symptom scores measured by Posttraumatic Stress Disorder Checklist for *DSM-5* (PCL-5). Initial: scores reported upon presentation to the emergency department. Completers: participants who completed all 3 RISE Guide modules (n=16). Incompleters: participants who completed less than 3 RISE Guide modules. Control: participants randomized to the control condition. Cutoff: dotted line indicates the PCL-5 cutoff score of 45. DSM-5: Diagnostic and Statistical Manual of Mental Disorders, Fifth Edition; RISE: Randomized Controlled Trial for Innovating Stress-Related E-Health.

Multiple imputation was used to account for missing data at later time points. At week 1 prior to randomization, all participants randomized to the RISE Guide reported clinically significant and elevated PTSD symptoms (mean 57.56, SD 11.96), which decreased at week 7 (mean 52.75, SD 15.83; Figure 2). From week 7 to month 6, PTSD scores continued to decrease (mean 44.11, SD 17.09). Between week 1 and month 6, there was a 13-point decrease in PTSD symptoms according to 2-tailed paired *t* tests (Hedge *g*=0.84), reflecting clinically significant change [36]. However, a between-group linear regression analysis indicated that the RISE Guide condition was not a significant predictor of reductions in PTSD symptoms (*P*=.90).

### RISE Guide Completers

#### Anxiety Sensitivity

**Figure 3.**
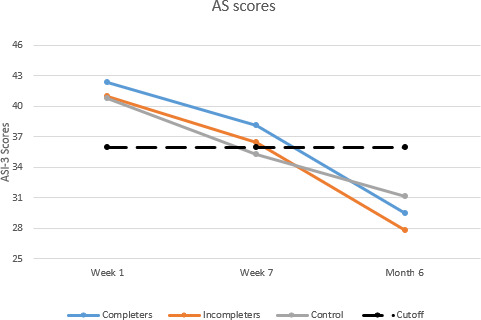
Anxiety sensitivity (AS) score changes among women sexual assault survivors presenting for emergency care participating in the randomized controlled trial for posttraumatic stress disorder prevention*.* ASI: anxiety sensitivity as measured by Anxiety Sensitivity Index-3 (ASI-3). Initial: scores reported upon presentation to the emergency department. Incompleters: participants who completed less than 3 RISE Guide modules. Control: participants randomized to the control condition. Completers: participants who completed all 3 RISE Guide modules (n=16). Cutoff indicates ASI-3 cutoff score of 36 (moderate anxiety sensitivity). RISE: Randomized Controlled Trial for Innovating Stress-Related E-Health.

Per-protocol analyses were also conducted among individuals who completed the RISE Guide intervention (defined as individuals who completed all three modules, n=16). Prior to randomization, at week 1 post sexual assault, AS scores exceeded the clinical cutoff (mean 42.38, SD 16.37; Figure 3), which decreased by week 7 (mean 38.13, SD 16.72). From week 7 to month 6, AS scores decreased though not significantly (mean 29.46, SD 17.86). According to a 2-tailed paired *t* test with multiple imputation, by month 6, the RISE Guide completers reported a 15-point reduction in AS (Hedge *g*=0.81).

#### Posttraumatic Stress Disorder

At week 1 post sexual assault and before randomization, PTSD scores were clinically significant and elevated (mean 56.19, SD 14.06; Figure 2). After the completion of the RISE Guide at week 7, there was a decrease in PTSD symptoms (mean 42.27, SD 16.28). At month 6, participants reported a decrease in PTSD scores (mean 40.62, SD 20.64). Overall, a 2-tailed paired *t* test with multiple imputation revealed that there was an 18-point reduction in PTSD symptom scores (Hedge *g*=0.80), reflecting clinically significant change [36].

### Acceptability of Control Intervention

To assess treatment acceptability, individuals randomized to the B2R condition completed the CEQ. Individuals randomized to the B2R condition reported slightly lower acceptability scores compared to the RISE Guide (mean 28.56, SD 16.87).

### Impact of Control Intervention on AS

Multiple imputation was used to account for missing data at later time points. Among participants randomized to the control condition, there was a decrease in AS between week 1 (mean 40.91, SD 17.91) and week 7 (mean 35.67, SD 16.98). Between week 7 and month 6, there were decreases in AS scores (mean 29.90, SD 17.01). Overall, from week 1 to month 6, there was an 11-point reduction in AS (Hedge *g*=0.61).

### Impact of Control Intervention on PTSD Symptoms

Multiple imputation was used to account for missing data at later time points. At week 1 post sexual assault, participants randomized to the control condition reported PTSD symptom scores exceeding the clinical cutoff (mean 57.74, SD 11.90). By week 7, participants reported decreases in their PTSD symptom scores (mean 49.04, SD 17.43). At month 6, participants reported a decrease in PTSD symptom scores (mean 44.94, SD 14.87). Between week 1 and month 6, a 2-tailed paired *t* test indicated there was a 13-point reduction in PTSD symptom scores (Hedge *g*=0.73), indicating clinically significant change [36].

### Feasibility of the RCT

#### Recruitment

In line with recruitment goals, on average, 3 (SD 2.70) participants were enrolled per month.

#### Retention

At week 7, 45 (75%) participants completed the survey, while 12 (20%) did not complete it. At month 6, 36 (60%) participants completed their surveys and 16 (26.7%) did not. No adverse events were reported during the study. The results indicate that, at week 7, retention was greater than or equal to the goal rate of 70%.

#### Intervention Completion

In the RISE Guide intervention, 15 of 27 (55.6%) participants completed all 3 modules on time and in full, while 1 of 27 (3.7%) completed the intervention in full but not on time. Eleven of the 27 (55.6%) participants randomized to the RISE Guide did not complete all 3 of the RISE Guide modules. Of those 11, 2 (7.4%) participants completed 1 of the 3 modules. In total, 9 of 27 (33.3%) randomized to the RISE Guide participants did not complete any modules. Almost half of participants assigned to the RISE Guide completed all modules on time and in full, which was less than the desired 70% completion rate.

#### EMA Adherence Rates

Adherence rates were measured based on the number of EMAs completed out of a total of 126. Excluding participants who did not initiate the EMAs at all, or who started them more than 14 days after the EMA period started (n=4), on average, participants completed 51.2% (6222/12,152; mean 64.5, SD 48.6) of the total EMAs. The percentage of the total EMAs completed ranged from 5.6% (7 EMAs) to 127.8% (161 EMAs; >100% is possible because participants received 4 surveys per day and were only required to complete 3).

## Discussion

The current phase 2b pilot randomized study of 60 women sexual assault survivors presenting to emergency care tested (1) the replication of acceptability, (2) the proof of concept of a novel smartphone-based intervention targeting AS (RISE Guide) in reducing AS and PTSD symptoms, (3) the adequacy of a relaxation control condition (B2R), and (4) feasibility of conducting future efficacy-focused RCTs. As expected, given the high-risk nature of sexual assault and the elevated AS in the development of PTSD symptoms [8,12,37,38], this was a vulnerable sample with 88.8% (40/45) meeting the clinical cutoff of 33 on the PCL-5 for likely PTSD 7 weeks post assault and 80.6% (29/36) continuing to meet this cutoff at month 6 [30]. These rates are consistent with previous research, including large observational cohort studies [2,39]. Overall, individuals receiving the RISE Guide rated it as acceptable and were observed to have medium-to-large reductions in AS and PTSD by month 6, particularly for those who completed the intervention. However, similar effects were found for those randomized to B2R, and we were unable to track how much participants engaged in B2R, limiting our ability to draw conclusions. Recruitment and retention were found to be feasible; however, there were concerns regarding intervention completion for those randomized to the RISE Guide. There were also major concerns about the appropriateness of the control condition, given that it produced larger effects on AS and PTSD symptoms than expected.

With regard to replicating the acceptability and proof of concept of the RISE Guide, as expected, the results indicated the RISE Guide was rated as acceptable via a validated measure (the CEQ) [35] and a study-specific measure. This is consistent with prior research indicating the acceptability of digital AS interventions generally [18], as well as the RISE Guide intervention specifically [19]. Replicating prior research [19], the results indicated that the RISE Guide was associated with medium-to-large reductions in AS and PTSD symptoms by month 6. However, in comparison to our initial trial, participants in this study experienced smaller and nonsignificant reductions in symptoms at week 7. By month 6, the mean PCL-5 score was approximately 44 (clinically significant according to lower cutoffs of 33, but not higher cutoffs of 45) [30,32]. Reductions were also clinically significant (including those in the control condition). We examined per-protocol analyses, given that intervention completion was lower than expected, and found that reductions in AS and PTSD symptoms were relatively larger in the group that completed the intervention versus those who did not. Yet, it must be acknowledged that (1) individuals who complete behavioral interventions may be more motivated and thus more likely to change regardless of intervention content and (2) the natural recovery of PTSD symptoms is to be expected for many sexual assault survivors [37]. Therefore, these analyses cannot pinpoint whether the intervention itself caused symptom reductions or whether they were related to individual traits or natural recovery. Furthermore, given that significant effects were not found immediately post intervention, we cannot determine whether the intervention took longer than expected to impact outcomes, or if by month 6, natural recovery had occurred and thus accounted for the significant reductions found. This confound highlights the need for future fully powered RCTs that can rule out recovery effects as an explanatory factor for symptom reductions.

Regarding feasibility, the results suggested that recruitment was feasible, with 3 participants enrolled per month. This was in line with study goals. Retention was also feasible, with at least 60% (37/62) completing all follow-ups, including month 6. This is notable considering that retention rates within samples of women sexual assault survivors presenting for emergency care have been particularly challenging, with rates often ranging from about 50% to 70% [40,41]. EMA adherence rates were about 50%, suggesting the EMA protocol may have been burdensome for participants to complete. Furthermore, there was a large range of compliance, with some participants completing very few and some demonstrating nearly perfect compliance. To place this adherence rate in context, relatively lower EMA adherence rates have been found in prior research among sexual assault survivors [42]. However, about 50% adherence is somewhat disappointing and should be improved in future trials.

As hypothesized and necessary for adequate control conditions [43], participants also rated the control condition (B2R) as acceptable. In contrast to the hypothesis, however, and problematic for an efficacy trial, B2R participants also experienced medium-to-large reductions in AS. Although effect sizes were relatively smaller than in the RISE Guide condition, they were still medium-to-large and larger than desired for a control condition. B2R was selected as a control condition, as we hypothesized that it would result in reductions in PTSD, but to a lesser extent than the active condition [44]. We did not hypothesize that it would significantly change AS, but the current findings suggest that it did. However, it must be noted again that we cannot rule out the effects of natural recovery on reductions in AS. Even with this in mind, the current results suggest B2R may not be an appropriate control condition. In particular, it is difficult to estimate how many women in our sample would be expected to develop PTSD without any treatment, as there are no estimates to our knowledge available for how many women sexual assault survivors with high AS develop PTSD. The current sample demonstrated high rates of PTSD across both conditions, with 80.6% (29/36) continuing to meet criteria for probable PTSD at month 6. This is similar to an unselected sexual assault survivor sample [2,39] in an observational study, making it unclear whether our intervention was ineffective or if this group of women with high AS would have had even higher rates of PTSD without intervention. While the severity of PTSD symptoms highlights the importance of preventative interventions in this group, it also makes it difficult to assess intervention success without a benchmark of recovery rates in this high-risk group. Furthermore, there is currently no standard intervention provided at the time of emergency care to sexual assault survivors to prevent PTSD [45]. Thus, the inclusion of an active control is not aligned with the actual clinical experience of sexual assault survivors seeking emergency care, who do not systematically receive any active intervention for PTSD.

Given these issues, it may be appropriate to consider “treatment-as-usual” as a control condition in future studies. Current guidelines for SANE examinations require trauma-informed care, informed consent, a comprehensive physical assessment, evidence collection, medical care (eg, prophylactic treatment for sexually transmitted infections and pregnancy), and documentation. Currently, unlike physical needs, there is no standardized system of mental health risk assessment and prevention as part of the examination; however, SANEs must provide trauma-informed care, ensure physical or psychological safety, and provide referrals to appropriate follow-up care [46]. Using treatment-as-usual as a control would reflect the clinical reality on the ground, allow the collection of estimates of natural recovery among women sexual assault survivors with high AS, and better enable testing of whether our novel intervention holds promise for its intended use in emergency care. Indeed, experts have raised concerns about the selection of overly stringent and unrealistic active control conditions, particularly in the early phases of research, as these can (potentially inaccurately) reduce enthusiasm about promising novel interventions [43]. Treatment-as-usual would also appropriately reflect a relatively less formidable control that is appropriate for early-stage RCTs, such as this work.

In terms of active intervention feasibility, one-third of the participants did not initiate any component of the RISE Guide intervention. On the other hand, 59% (16/27) completed the entire intervention. This was consistent with our initial trial, in which 25% did not initiate the intervention [19]. It is expected that not all participants will complete behavioral interventions [47], given the motivation, time, and access to logistics required. Indeed, traditional interventions, such as cognitive processing therapy or prolonged exposure, have widely varying completion rates, which are often found to be around 40% to 50% [48-50]. Completion rates are particularly poor for digital health interventions, which tend to have low levels of engagement [51]. Given this context, the current completion rate of nearly 60% (16/27) is promising and within the range of gold-standard interventions. However, 33% (9/27) of the participants not initiating the intervention is concerning in the context of an RCT and may lead to problems with hypothesis testing in an efficacy trial. One way to overcome problems with intervention adherence is to ensure we are targeting the appropriate audience for our intervention [47]. A common approach to doing so is the addition of a “behavioral run-in” period mimicking what participants would be asked to do during the intervention, and only randomizing individuals who are able to successfully complete the behavioral run-in period [47]. Therefore, we suggest that future research consider using a behavioral run-in period similar to the intervention procedures, such as completing daily phone-based tasks, in order to ensure the appropriate target population for the intervention.

Overall, this study aligns with previous findings that cognitive behavioral secondary preventative interventions *may* be beneficial in mitigating PTSD after sexual assault. However, this is a challenging area in which proper RCT design is critical, given the natural recovery observed after trauma exposure. Specifically, future research should continue to test this potentially promising intervention by selecting a more appropriate control condition, such as treatment-as-usual, to better approximate the current clinical reality and allow for the establishment of typical rates of recovery in high-risk samples. Furthermore, using a behavioral run-in period to help ensure that the correct target sample is identified and treatment engagement is sufficiently high may be helpful. The results suggest that individuals with high AS are a vulnerable group after sexual assault, but it remains unclear whether targeting AS will lead to reductions in PTSD symptoms after sexual assault.

The results of the current study should be considered in light of its strengths and limitations. Strengths include that the RISE Guide was tested in the target population, and intervention completion was assessed objectively. Limitations include, first, as discussed, about a third of those randomized to the RISE Guide did not initiate the treatment. Second, we were also unable to assess how many of those randomized to B2R initiated or completed that intervention, so it is unclear how these rates compare. Therefore, we are unable to interpret any between-group comparisons or comment on the feasibility of using B2R as a control condition in future work. Third, although consistent with a phase 2b trial [21], the sample size was relatively homogeneous and limited. As research progresses, larger and more diverse samples (eg, men and gender diverse sexual assault survivors) should be included. Fourth, larger sample sizes would also enable direct statistical comparisons between the active and control groups’ clinical outcomes. Fifth, larger sample sizes would allow for the calculation of more reliable effect sizes. Effect sizes derived from pilot RCTs such as this one should be interpreted with caution [24].

This pilot study of 60 women sexual assault survivors tested the feasibility and replicated the proof of concept of a novel smartphone-based intervention targeting AS versus a relaxation control to reduce AS and, in turn, PTSD symptoms. The findings reinforced the vulnerability of this population, with high rates of PTSD observed 6 months after the sexual assault. Acceptability and proof-of-concept findings replicated prior research [19] and suggest that more research is needed, particularly given the larger than expected effects of the control condition and the need to rule out recovery effects. This work is essential to the development of effective scalable early interventions for this common trauma population that experiences great suffering.

## Supplementary material

10.2196/86612 Multimedia Appendix 1 Zero-order correlation table for anxiety sensitivity and posttraumatic stress disorder symptoms among women sexual assault survivors.

10.2196/86612 Checklist 1 CONSORT checklist.

## Abbreviations

AS: anxiety sensitivity
ASI-3: Anxiety Sensitivity Index-3
B2R: Breathe2Relax
CEQ: Credibility/Expectancy Questionnaire
*DSM-5*: *Diagnostic and Statistical Manual of Mental Disorders, Fifth Edition*
EMA: ecological momentary assessment
EMI: ecological momentary intervention
PCL-5: PTSD Checklist-5
PTSD: posttraumatic stress disorder
RA: research assistant
RCT: randomized controlled trial
REDCap: Research Electronic Data Capture
RISE: RCT for Innovating Stress-Related E-Health
SANE: sexual assault nurse examiner
UNC: University of North Carolina
