# Supplementary material for: Testing a Smartphone-Based Intervention Targeting Anxiety Sensitivity Among Women Presenting for Emergency Care After Sexual Assault: Pilot Randomized Controlled Trial
Source: JMIR Form Res. 2026 May 25;10:e86612. doi: 10.2196/86612 (PMC13201896; doi:10.2196/86612)
Supplement: Multimedia Appendix 1 [file formative-v10-e86612-s001.docx]

**Multimedia Appendix 1**

**Supplemental Table 1**

Zero-Order Correlation Table for AS and PTSD Symptoms among Women Sexual Assault Survivors Presenting for Emergency Care Participating in the Present Randomized Controlled Trial for Posttraumatic Stress Disorder Prevention

|  | 1 | 2 | 3 | 4 | 5 | 6 | 7 | 8 | 9 | 10 |
| --- | --- | --- | --- | --- | --- | --- | --- | --- | --- | --- |
| 1. Week-1 AS | - | - | - | - | - | - | - | - | - | - |
| 1. Week-1 PTSD | .44*** | - | - | - | - | - | - | - | - | - |
| 1. Residualized Changes in AS (Week-1 to Week-7) | -- | .10 | - | - | - | - | - | - | - | - |
| 1. Residualized Changes in PTSD (Week-1 to Week-7) | .01 | -- | .36* | - | - | - | - | - | - | - |
| 1. Week-7 AS | .73*** | .36* | .68*** | .26 | - | - | - | - | - | - |
| 1. Week-7 PTSD | .19 | .46** | .37*** | .89*** | .39** | - | - | - | - | - |
| 1. Residualized Changes in AS (Week-7 to Month-6) | .34* | .15 | -.11 | <.01 | .16 | .07 | - | - | - | - |
| 1. Residualized Changes in PTSD (Week-7 to Month-6) | .46** | .41* | -.06 | -.11 | .29 | .10 | .72*** | - | - | - |
| 1. Month-6 AS | .66*** | .31 | .41* | .13 | .78*** | .12 | .75*** | .65*** | - | - |
| 1. Month-6 PTSD | .41* | .44** | -.02 | -.02 | .32 | .20 | .71*** | .99*** | .66*** | - |

*Note*: AS = Anxiety Sensitivity; PTSD = Posttraumatic Stress Disorder Symptoms; * = *p* < .05, ** = *p* < .01, *** = *p* < .001. Residualized change scores were calculated using unstandardized regression-based residuals.
